# Advanced Ovarian Pregnancy: A Case Report of Misdiagnosis and Surgical Outcomes

**DOI:** 10.1155/2022/8856078

**Published:** 2022-12-16

**Authors:** Noppamart Whangteeranon, Pang Pinyochotiwong

**Affiliations:** ^1^Department of Obstetrics and Gynecology, Bang Saphan Hospital, Prachuap Khiri Khan, Ministry of Public Health, Thailand; ^2^Department of Radiology, Bang Saphan Hospital, Prachuap Khiri Khan, Ministry of Public Health, Thailand

## Abstract

**Background:**

Ovarian pregnancy is rare and difficult to diagnose preoperatively, especially in advanced gestational age. Misdiagnosis can increase the chance of emergent complications.

**Case:**

A misdiagnosed case of a 33-week ovarian pregnancy woman who underwent surgery due to a preoperative diagnosis of a dead fetus in utero, and transverse lie was reported with eventful surgical outcomes. This case resulted in the delivery of a dead fetus and a sudden massive hemorrhage that occurred after placental detachment. Oophorectomy could not be performed due to severe bowel adhesions and multiple feeding vessels from the bowel at the posterior part of the left ovary. The placental site was sutured at multiple sites, and local hemostatic agents were placed for hemostasis. Postoperatively, she received multiple transfusions and was safely discharged after 10 days.

**Conclusion:**

Obstetricians must be acutely aware of this condition, especially while performing sonography preoperatively, for better outcomes.

## 1. Introduction

Ovarian pregnancy is a rare condition with an incidence of approximately 1/7,000–1/40,000 and is difficult to diagnose preoperatively in advanced gestational age [1, 2]. Misdiagnosis can increase the chance of emergent complications. Preoperative preparation, such as blood crossmatching, or referral to a more specialized hospital should be performed for better results.

## 2. Case Presentation

**Figure 1:**
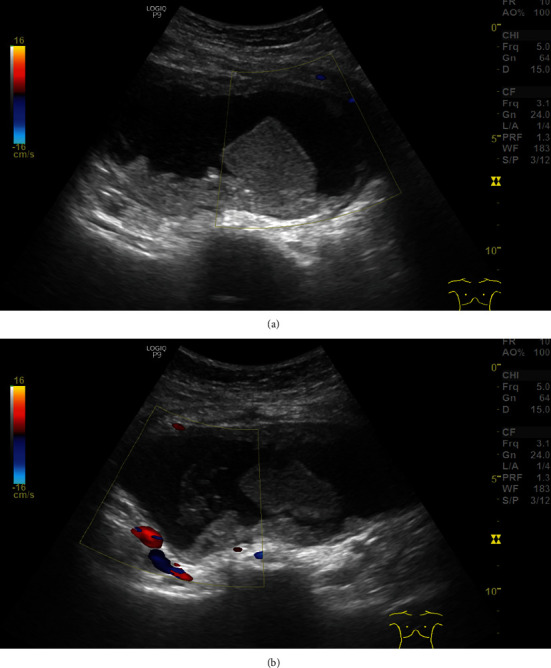
Ultrasound images of persistent left ovarian cyst (3 months later). A hypoechoic cyst with a few polygonal shape density lesions inside the ovarian area with the hypogastric vessel as a landmark was shown on the images. (a) A hypoechoic cyst with a few polygonal shape lesion inside. (b) The hypogastric vessel as a landmark of the ovary.

**Figure 2:**
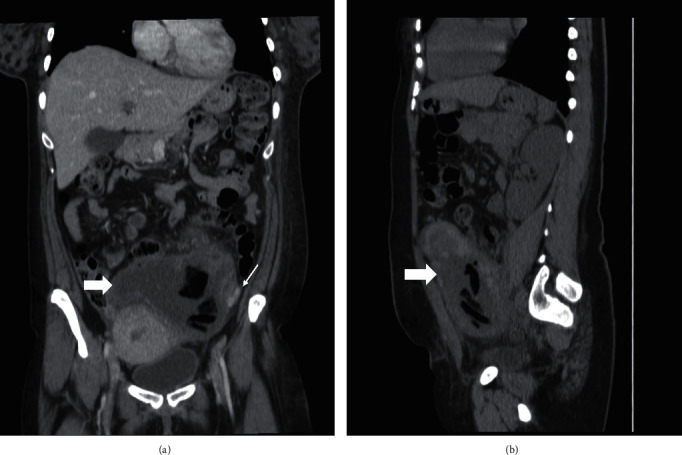
Computed tomography images of the left ovarian cyst (thick arrow) (3 months later). Large heteroechoic cystic lesion that drained to the left enlarged ovarian vein (thin arrow) with several bizarre shapes, low density lesions inside the left ovary, as shown on (a) coronal section and (b) sagittal section.

**Figure 3:**
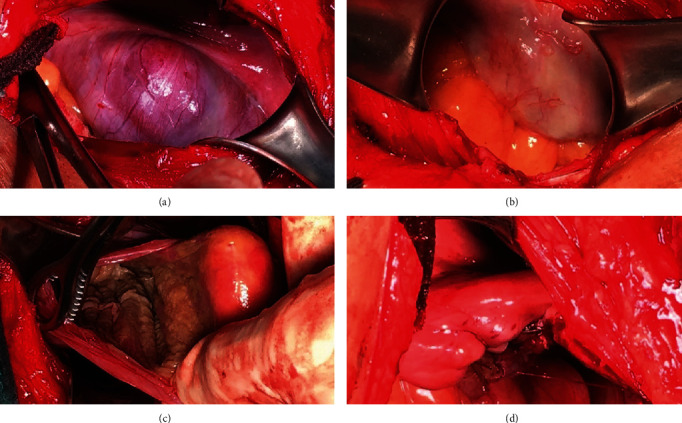
Intraoperative finding in the second laparotomy (13 months later). (a) Intact left ovarian wall with ovarian ligament proper. (b) Bowel adhesion blended around the left ovary. (c) Internal surface of the left ovarian cyst. (d) Postmarsupialization of the ovarian wall.

A 34-year-old woman with gravida 2, parity 1, at 33 weeks 6 days pregnancy who had antenatal care with a midwife and passed an ultrasound scan once in the second trimester by a general practitioner was presented. Her previous delivery was spontaneous vaginal delivery at term. She presented at the provincial hospital with preterm labor, fetal growth restriction, and fetal distress. Finally, the fetus died before the emergency operation was performed, so she was referred to a community hospital for termination of pregnancy. She did not have abdominal pain, vaginal bleeding, or leakage. Her vital signs and general appearance were unremarkable. Her physical examination showed a soft nondistended abdomen and no uterine contraction with a small fundal height for gestational age. Her pelvic exam was performed by a midwife, and no cervical dilatation was found. The obstetrician performed an ultrasonogram preoperatively, which was the first sonogram performed by a specialist and mistook her uterus for a myoma. Therefore, she was scheduled for cesarean delivery due to a preoperative diagnosis of a dead fetus in utero with a transverse lie. In the operative field, after gaining access to the abdominal cavity, no blood or blood clots were detected as in the usual cesarean section case. The ovary, which was thought to be the uterus, looked strange with slightly paler, smoother, and thinner than typical uterus. Furthermore, examination was limited due to the Pfannenstiel incision. A transverse incision and blunt dissection were performed to open the ovary. The intact amniotic sac was found and then punctured to deliver a dead fetus weighing 1,525 g with minor anomalies including ambiguous genitalia, cleft lip, and small ear defects. While an entire placenta was easily removed, the ovary wall nearly inversed inside out and massive hemorrhage suddenly occurred. The operative field was full of blood even though continuous suction was performed. Therefore, multiple packed swabs were used for pressure instead. After approximately 5 minutes of pressure, the rate of bleeding was slower, and the field was visualized. The surgeon realized that it might be a left ovarian ectopic pregnancy because of its white spherical shape attached to the lateral side of the normal size uterus with the ovarian ligament proper. No placental tissue was identified outside that ovary. The ipsilateral tube was intact and elongated. Oophorectomy was attempted but could not be done because all posterior parts of the 20 cm size ovary adhered to bowel loops with large multiple feeding vessels from them. In addition, bowel resection is not possible in a community hospital without a general surgeon. Even after multiple sutures were placed at the raw surface inside the ovary, the bleeding continued. Many pieces of local hemostatic agents were packed to control the bleeding. After that, the ovarian incision was continuously sutured, and the abdominal wall was closed layer by layer. Three units of packed red cells and two units of fresh frozen plasma were transfused. The estimated total blood loss was 1,800 ml. She had a fever and signs of anemia, during the first night postoperatively and had a full recovery after 10 days of admission. The pathology report of the placenta was normal placenta without ovarian tissue. She had regular follow-ups and found a persistent 12-cm left ovarian cyst, which might be a fluid collection or old hematoma at three months. The size gradually decreased but persisted at 10 cm for more than a year with occasional mild pain (Figures 1 and 2). Therefore, a second look operation was performed, the left ovarian cyst was anatomically identified, and oophorectomy could not be performed for the same reason. There was serous fluid with remains of multiple pieces of local hemostatic agent inside. The left ovary was opened, drained, and marsupialized to decrease recurrence (Figure 3). The estimated blood loss was 100 ml. No surgical complications or recurrence was detected within 1 year.

## 3. Discussion

An ovarian ectopic pregnancy usually ruptures and is terminated during the first trimester, with 5.3% proceeding to the second trimester and 3.7% to the third trimester [2]. Late ovarian pregnancy is rare and easy to misdiagnose especially in a case without a previous ultrasound scan by a specialist in the first trimester [3, 4]. In this case, while the ultrasound was focused on the fetus including weight, lie, heart activity, amniotic fluid, and placental location, the continuity of structure around the gestational sac to cervix was not observed. Moreover, the true uterus was thought to be a myoma causing an abnormal lie of the fetus. Complete physical and pelvic examination, preoperative ultrasound scan with great attention, and keeping in mind of advance ovarian pregnancy may make the correct diagnosis and aid in preoperative preparation.

Fetal prognoses of late ovarian pregnancy are mostly dead, but many cases of advanced ovarian pregnancy with a surviving fetus have also been reported [1, 3, 5, 6]. The fetus in this case was preterm, lying in a transverse position to the mother, and had minor anomalies, similar to other reports [3, 5]. The pathology report did not note the placenta present in ovarian tissue and, thus, did not fulfill Spiegelberg's criteria for ovarian pregnancy. It could be a secondary ovarian pregnancy or extrafollicular type of both primary and secondary ovarian pregnancy [7]. The diagnosis in this case was made on multiple findings in the operative field, confirmed with postoperative imaging and the second-look operation [8].

There are complications of an ovarian ectopic pregnancy, including intraabdominal hemorrhage from a ruptured sac, hemodynamic instability, and massive hemorrhage from the implantation site of the placenta [5]. Compared to some other reports, this case is the worst because of misdiagnosis, occurrence in a community hospital, and limitation of bowel surgery without a specialist [3, 5, 6, 9]. Bleeding from a placental implantation site can be life-threatening. Ligation of the cord to leave a placenta in situ is one method that leads to less bleeding but a higher risk of infection. In this case, the placenta was easily removed and resulted in massive hemorrhage, which made it very difficult to achieve hemostasis. The pressure was better than suction to visualize the operative field, and staying calm is one of the keys to achieving this.

The local hemostatic agent—Gelfoam®, which is an absorbable gelatin sponge, was used for emergencies in this case with no certain effect [10]. There are reports of using it as an adjuvant in placental site bleeding of the uterine cavity or postpartum hemorrhage cases and gynecological cases without complications, but it has not been reported in an ovarian pregnancy [11, 12]. Delayed absorption of the hemostatic agent for longer than a year has not been reported either [11, 13]. Nevertheless, no other chronic complication was detected in this case except the persistence of the ovarian cyst.

Early and accurate preoperative diagnosis is important to prevent emergency and adverse outcomes. Clinicians should be aware of this condition while performing ultrasound scans especially in cases of poor prenatal care or in a rural setting without a specialist.

## Data Availability

The references data used to support the findings of this study are included within the article.
